# Retropharyngeal abscess following a gun shot injury

**DOI:** 10.1016/S1808-8694(15)30559-0

**Published:** 2015-10-19

**Authors:** Shitij Arora, J.K. Sharma, S.K. Pippal, Abhinav Yadav, Murtaza Najmi, Deepanshu Singhal

**Affiliations:** 1 ENT, gandhi medial college bhopal india; 2 ENT, gandhi medical college bhopal india; 3 ENT, gandhi medical college bhopal india; 4 ENT, gandhi medial college bhopal india; 5 ENT, gandhi medial college bhopal india; 6 ENT, gandhi medial college bhopal india

**Keywords:** abscess, retropharyngeal, trauma

## INTRODUCTION

A retropharyngeal abscess is an infection in one of the deep spaces of the neck. An abscess in this location is an immediate life-threatening emergency with potential for airway compromise and other catastrophic complications.

In adults, retropharyngeal abscess can occur as a result of local trauma such as foreign body ingestion like fishbones^1^ or instrumental procedures like laryngoscopy, endotracheal intubation, surgery, endoscopy, feeding tube placement and odontogenic infections.^2^

There are very few reported cases of a retropharyngeal abscess following gun shot injury and the special feature in this case was that there was no retained foreign body in the retropharyngeal space.

## CASE REPORT

A 17 yr old male presented at the Department of ENT Gandhi Medical College, Bhopal with complaints of pain in throat and difficulty in swallowing since 3 days. Patient gave history of gunshot over neck 3 days back.

Local examination of neck was done. Gunshot entry wound was present on right side of neck 2 cm away from midline just below the hyoid bone. Wound was surrounded by black eschar. There was diffuse swelling and tenderness around the wound. An exit wound was present on left side of neck approximately 1cm behind the posterior border of sternocleidomastoid muscle.

On oropharyngeal exam posterior pharyngeal wall was congested and a diffuse swelling was present involving both sides of midline. No other abnormality was detected.

**Figure 1:**
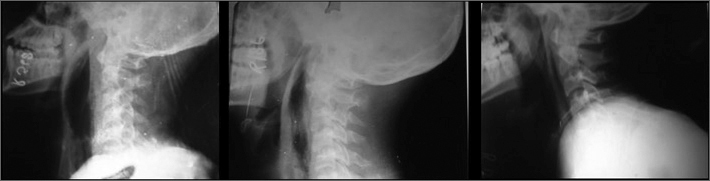
sequential lateral neck x-rays showing resolution of the abscess

On indirect laryngoscopic examination, epiglottis was congested and rest of the structures are normal. Widening of the prevertebral space was seen in X-ray. Sequential X rays were taken till the resolution of the abscess (fig 1). There was no evidence of any pellet or foreign body density in the retropharyngeal area and CT scan was done to rule out the same confirming × ray findings.

Aspiration from retropharyngeal space was done and frank pus was aspirated. After that patient was posted for incision and drainage transorally under general anaesthesia and elective tracheostomy was done prior to drainage. The outcome of the patient was excellent. No complications were observed.

## DISCUSSION

The retropharyngeal space is bordered anteriorly by the constrictor muscles of the neck and posteriorly by prevertebral fascia that covers the musculus longissimus capitis and the musculus longissimus colli overlying the cervical vertebral bodies.^3^

In adults abscess due to naso-oropharyngeal infection is rare and is usually secondary to trauma, foreign bodies, or as a complication of dental infections. The organisms found in the pus in acute cases are staphylococcus aureus, streptococcus viridans, klebsiella pneumoniae, escherichia coli and heamophilus species.^3^, ^4^, ^5^

Very few cases of retropharyngeal abscess following gunshot injury have been reprorted so far, though cases have been reported following traumatic endotracheal intubation.

In this particular case there was history of gunshot over neck with pain in throat and difficulty in swallowing. Plain radiographic and CT scan showed widened prevertebral space and abscess respectively. Needle aspiration was done in operation theatre. Frank pus was aspirated. Intraoral incision and drainage was done with elective tracheostomy. Patient was relieved.
